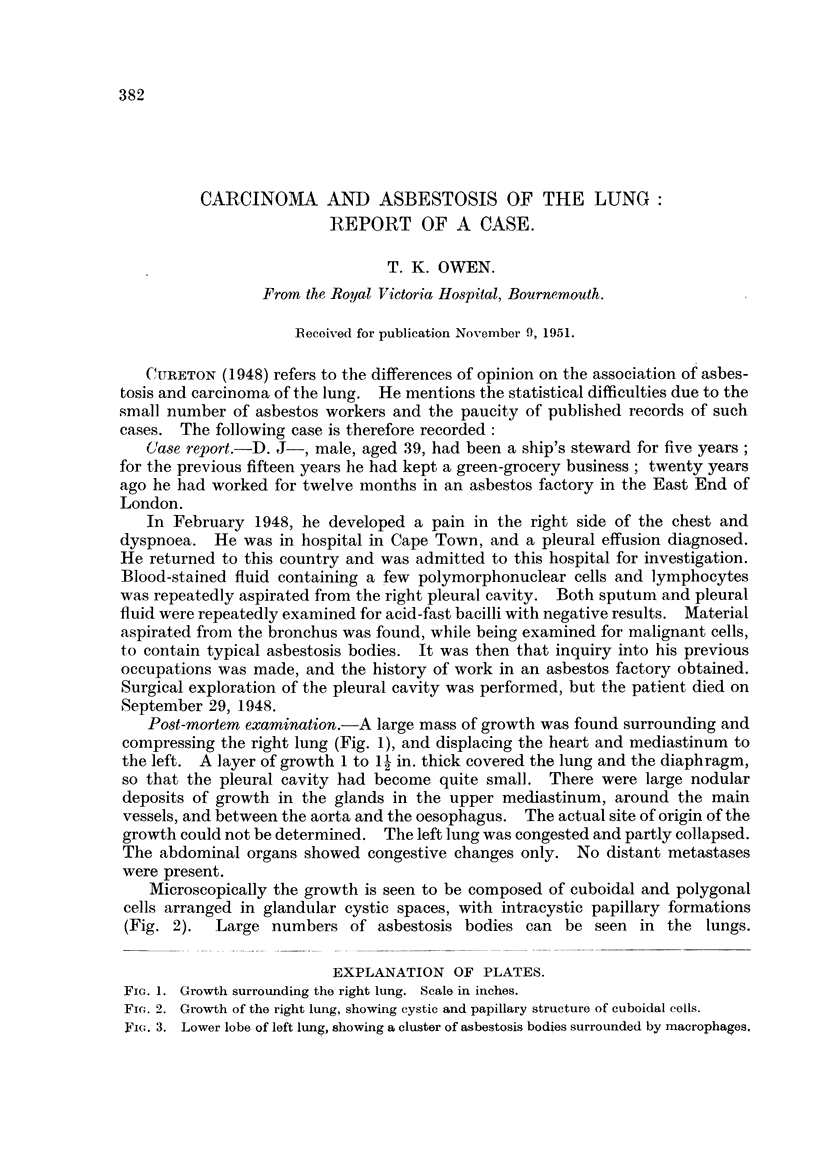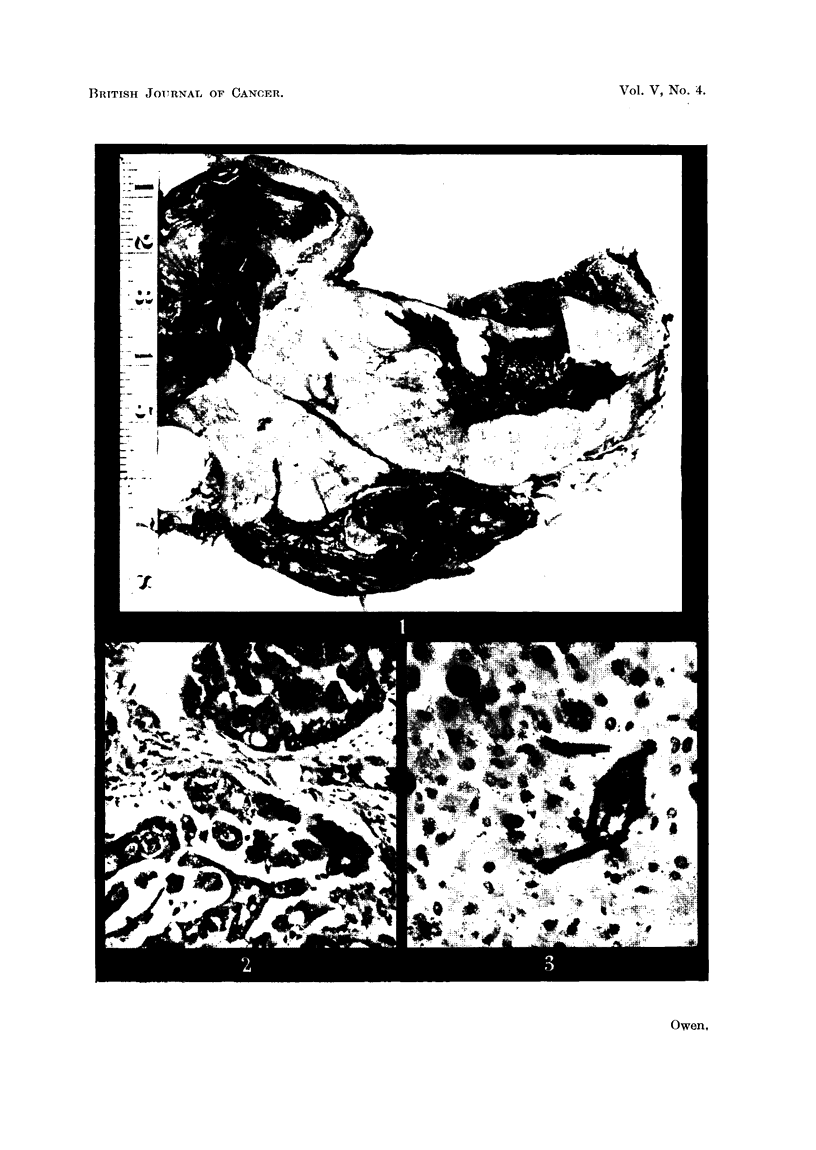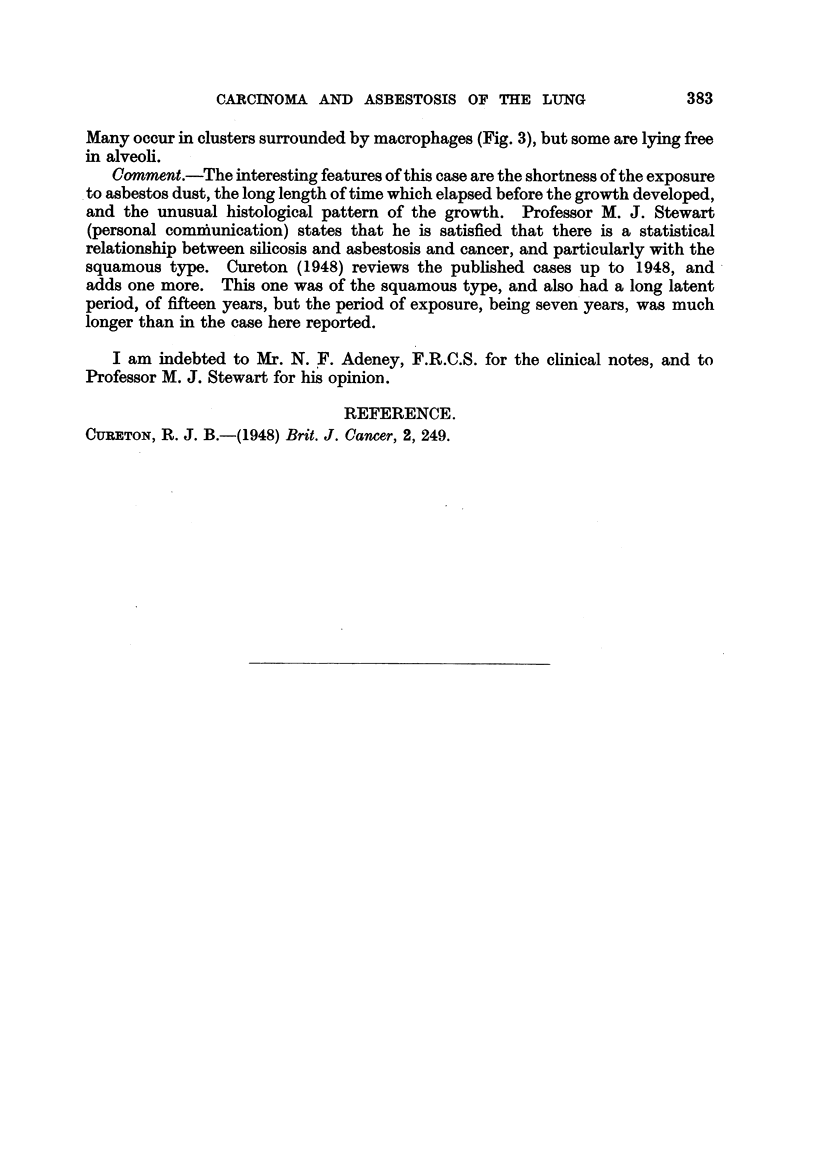# Carcinoma and Asbestosis of the Lung: Report of a Case

**DOI:** 10.1038/bjc.1951.43

**Published:** 1951-12

**Authors:** T. K. Owen

## Abstract

**Images:**


### CARCINOMA AND ASBESTOSIS OF THE LUNG: REPORT OF A CASE.

T. K. OWEN.

*From the Royal Victoria Hospital, Bournemouth.*

Received for publication November 9, 1951.

CURETON (1948) refers to the differences of opinion on the association of asbestosis and carcinoma of the lung. He mentions the statistical difficulties due to the small number of asbestos workers and the paucity of published records of such cases. The following case is therefore recorded :

*Case report.*—D. J—, male, aged 39, had been a ship's steward for five years ; for the previous fifteen years he had kept a green-grocery business ; twenty years ago he had worked for twelve months in an asbestos factory in the East End of London.

In February 1948, he developed a pain in the right side of the chest and dyspnoea. He was in hospital in Cape Town, and a pleural effusion diagnosed. He returned to this country and was admitted to this hospital for investigation. Blood-stained fluid containing a few polymorphonuclear cells and lymphocytes was repeatedly aspirated from the right pleural cavity. Both sputum and pleural fluid were repeatedly examined for acid-fast bacilli with negative results. Material aspirated from the bronchus was found, while being examined for malignant cells, to contain typical asbestosis bodies. It was then that inquiry into his previous occupations was made, and the history of work in an asbestos factory obtained. Surgical exploration of the pleural cavity was performed, but the patient died on September 29, 1948.

*Post-mortem examination.*—A large mass of growth was found surrounding and compressing the right lung (Fig. 1), and displacing the heart and mediastinum to the left. A layer of growth 1 to $1\frac{1}{2}$ in. thick covered the lung and the diaphragm, so that the pleural cavity had become quite small. There were large nodular deposits of growth in the glands in the upper mediastinum, around the main vessels, and between the aorta and the oesophagus. The actual site of origin of the growth could not be determined. The left lung was congested and partly collapsed. The abdominal organs showed congestive changes only. No distant metastases were present.

Microscopically the growth is seen to be composed of cuboidal and polygonal cells arranged in glandular cystic spaces, with intracystic papillary formations (Fig. 2). Large numbers of asbestosis bodies can be seen in the lungs.

EXPLANATION OF PLATES.

FIG. 1. Growth surrounding the right lung. Scale in inches.

FIG. 2. Growth of the right lung, showing cystic and papillary structure of cuboidal cells.

FIG. 3. Lower lobe of left lung, showing a cluster of asbestosis bodies surrounded by macrophages,

1

2

3

Owen.

Many occur in clusters surrounded by macrophages (Fig. 3), but some are lying free in alveoli.

*Comment.*—The interesting features of this case are the shortness of the exposure to asbestos dust, the long length of time which elapsed before the growth developed, and the unusual histological pattern of the growth. Professor M. J. Stewart (personal communication) states that he is satisfied that there is a statistical relationship between silicosis and asbestosis and cancer, and particularly with the squamous type. Cureton (1948) reviews the published cases up to 1948, and adds one more. This one was of the squamous type, and also had a long latent period, of fifteen years, but the period of exposure, being seven years, was much longer than in the case here reported.

I am indebted to Mr. N. F. Adeney, F.R.C.S. for the clinical notes, and to Professor M. J. Stewart for his opinion.

### REFERENCE.

CURETON, R. J. B.—(1948) *Brit. J. Cancer*, 2, 249.